# Staged Reconstruction Is Not Necessary Following Oncologic Resection of Superficial Myxofibrosarcoma

**DOI:** 10.3390/cancers17223637

**Published:** 2025-11-12

**Authors:** Leilani Garayua-Cruz, Samuel E. Broida, Mikaela H. Sullivan, Andrew L. Folpe, Meng X. Welliver, Katie N. Lee, Brittany L. Siontis, Steven I. Robinson, Thanh P. Ho, Scott H. Okuno, Peter S. Rose, Karim Bakri, Steven L. Moran, Matthew T. Houdek

**Affiliations:** 1 School of Medicine, University of Puerto Rico, San Juan, PR 00936, USA; 2 Department of Orthopedic Surgery, Mayo Clinic, Rochester, MN 55905, USA; 3 Department of Laboratory Medicine and Pathology, Mayo Clinic, Rochester, MN 55905, USA; 4 Department of Radiation Oncology, Mayo Clinic, Rochester, MN 55905, USA; 5 Department of Medical Oncology, Mayo Clinic, Rochester, MN 55905, USA; 6 Division of Plastic and Reconstructive Surgery, Mayo Clinic, Rochester, MN 55905, USA

**Keywords:** myxofibrosarcoma, VAC therapy, recurrence, staged reconstruction

## Abstract

Background: Myxofibrosarcomas are notoriously highly infiltrative soft-tissue sarcomas, making negative surgical margins difficult to obtain. Recently, vacuum-assisted closure (VAC) is used to delay wound closure until a negative margin has been achieved; however, this can delay care and increase costs. Our institution has historically performed single-stage resections with intraoperative frozen margin analysis and reconstruction in these patients. The purpose of this study is to report the outcomes of this technique. Methods: We reviewed 112 patients (62 males, mean age 70 ± 14 years) with superficial myxofibrosarcoma. Eighty-eight patients received preoperative radiation. All patients underwent surgical resection with intraoperative frozen margin analysis, and the planned reconstruction was performed in a single anesthetic. Results: The 10-year local recurrence-free survival was 90%; positive intraoperative frozen section (HR 7.44, *p* = 0.004) and final permanent margins (HR 8.53, *p* = 0.007) were associated with local recurrence. Intraoperative margins were negative in 103 (92%) of patients, 1 of which was positive on final permanent section. There were nine cases of microscopically positive margins, of which seven underwent immediate re-excision to a negative margin. The accuracy of frozen margin assessment for myxofibrosarcoma was between 92.92 and 98.23%. All patients underwent reconstruction at the time of resection, with 19% needing an additional procedure, most commonly due to a wound complication (12%). Conclusions: Multidisciplinary single-stage excision with intraoperative frozen margin assessment and soft-tissue reconstruction yields low rates of local recurrence in patients with superficial myxofibrosarcoma.

## Simple Summary

Myxofibrosarcomas are a common superficial soft-tissue sarcoma and are known for an infiltrative growth pattern along a fascial plane. To achieve a wide margin, large resections are often required, which need skin-grafting or soft-tissue coverage. Recent publications have advocated for staged resection and only reconstructing once the final permanent margin is negative to reduce the risk of local recurrence. This increases the cost of care and patient morbidity secondary to additional procedures. We have historically utilized a combination of wide local resection, with intraoperative frozen section analysis and immediate soft-tissue coverage, with a low rate of local recurrence.

## 1. Introduction

Soft-tissue sarcomas (STSs) account for less than 1% of all adult malignancies in the United States [1]. Among them, myxofibrosarcoma (MFS) is a relatively common subtype which often arises in the superficial soft tissues of the extremities or trunk [2,3]. MFS is notorious for its highly infiltrative growth pattern and for having the highest rates of local recurrence (LR), making complete surgical excision with negative margins the cornerstone of treatment [4,5]. However, achieving negative margins can be difficult due to the tumor’s propensity for microscopic extension along fascial planes, complicating intraoperative assessment [6,7].

Recently, centers have adopted a strategy of temporizing soft-tissue coverage using wound vacuum-assisted closure (VAC) devices, deferring definitive reconstruction until final pathologic evaluation confirms margin status. In these studies, the authors noted a lower rate of local recurrence and fewer re-excisions when the wound was temporized with a VAC dressing, compared to their historic practice of a single-stage excision [8]. Nevertheless, the use of VAC devices is not without complications, which include increased risk of infection, bleeding, prolonged hospital stays, and patient discomfort, which potentially impact recovery and quality of life [8,9,10,11].

At our institution, we rarely utilize VAC temporization and instead rely on a multidisciplinary team of orthopedic oncologists, plastic surgeons, and pathologists to utilize intraoperative frozen section analysis for all soft-tissue sarcomas. This allows for confirmation of margins during the tumor extirpation and planned immediate soft-tissue reconstruction. This study aims to assess the outcomes of patients with superficial MFS undergoing single-stage resection and reconstruction with frozen margin assessment at our institution.

## 2. Materials and Methods

Following approval from our institutional review board, we retrospectively reviewed 212 cases of histologically confirmed MFS treated at our institution between 1995 and 2023. The group included patients undergoing a work-up for a primary or recurrent tumor and those with a history of a previous non-oncologic resection. During this period, one patient presented with a fungating MFS which was temporized with a wound vac following resection to confirm final margins and was not included in the investigation. In addition, 99 patients with a tumor deep to the fascia were excluded. The remaining group included 112 (62 male; 50 female) patients with a mean age at the time of surgery of 70 ± 14 years (Table 1).

The diagnosis was made based on a needle biopsy (*n* = 32, 28%), incisional biopsy performed at an outside institution (*n* = 10, 9%), or following an inadvertent excision (*n* = 70, 63%). Tumors were in the lower extremity (*n* = 63, 56%), upper extremity (*n* = 41, 37%), and trunk (*n* = 8, 7%). Tumors were graded based on the Fédération Nationale des Centres de Lutte Contre le Cancer (FNCLCC) scoring system [12], comprising low-grade (*n* = 9, 8%), intermediate grade (*n* = 34, 30%), and high grade (*n* = 69, 62%).

All patients were treated in a multidisciplinary fashion. Radiotherapy was administered to 88 (79%) patients. Radiation was most frequently delivered with preoperative external beam radiotherapy (*n* = 68, mean total dose 48.9 ± 2.6 Gy). Additional radiotherapy techniques included postoperative external beam (*n* = 8, mean total dose 59.1 ± 7.4 Gy); preoperative external beam and intraoperative external beam (*n* = 7, mean total dose 58.9 ± 2.8 Gy); preoperative external beam and postoperative brachytherapy (*n* = 3, mean total dose 59 ± 8.3 Gy); brachytherapy and external beam radiotherapy (*n* = 1, total dose 65.4 Gy); and brachytherapy alone (*n* = 1, total dose 45 Gy). Twenty-four (21%) patients did not receive radiotherapy. Indications to forego radiotherapy included a small (<5 cm), low-grade, and superficial sarcoma with the ability to achieve wide margins without radiotherapy (*n* = 13); planned postoperative external beam radiotherapy, but the patient developed a wound complication or there was concern about a skin graft site (*n* = 6); amputation (*n* = 3); history of a sarcoma/cancer predilection syndrome (*n* = 1); or previous radiotherapy doses to the area (*n* = 1).

Chemotherapy was given to 8 patients (7%): most commonly, neoadjuvant mitomycin, doxorubicin, and cisplatin (*n* = 7) [13]. One additional patient received neoadjuvant mitomycin, doxorubicin, and cisplatin and adjuvant doxorubicin and ifosfamide. Chemotherapy was given as a radiosensitizer (*n* = 5) and due to concern for extensive soft-tissue contamination in the setting of an inadvertent excision (*n* = 3).

Following the completion of neoadjuvant treatments, all patients were evaluated by our plastic and reconstructive surgeons in conjunction with orthopedic oncology to develop a plan for immediate coverage and closure. All patients were treated surgically with the goal of achieving a negative margin on the resected specimen. For MFS, we often plan a minimum of 4–5 cm of radial margin to the palpable area of tumor or scar from a previous excision, taking the muscular fascia or periosteum as a deep margin. All specimens were reviewed prior to reconstruction with intraoperative frozen section analysis of the entire resected specimen to confirm negative margins. Scouting margins along the periphery of the tumor were not routinely taken.

All specimens were examined and prepared for frozen sectioning by taking multiple blocks of tissue from the margin. The frozen section technique utilized at Mayo Clinic Rochester, which differs somewhat from the technique utilized in most other institutions, has been previously described in detail [14]. Briefly, appropriately trimmed fresh tissue is placed on a fixed metal stage to which a small amount of water has been applied. The stage is cooled rapidly by circulating liquid freon, reaching −30° to −40 °C in roughly 15 s. A histology technician then cuts the tissue at the interface between the frozen and unfrozen tissue with a swinging microtome blade. The frozen tissue section is transferred from this blade onto a glass rod and immersed in a toluidine blue stain for several seconds, rinsed with clean water, and then rolled onto a glass slide and cover slipped with a water mount. This entire process takes less than 1 min. All the remaining tissue is submitted for fixation, routine processing, and staining and then evaluated on permanent sections the following day. Our frozen section practice is not subspecialized, although subspecialist sarcoma pathologists are available for immediate consultation in all instances.

If there was concern for a positive margin on frozen section analysis, an additional 1–2 cm of radial subcutaneous tissue and any residual fascia/epineurium/adventitia/periosteum was taken in that area, if possible, based on where the margin was deemed positive, and the new margin was evaluated using frozen section analysis. The mean tumor size and volume on the resected specimen was 6 ± 4 cm and 131 ± 262 cm^3^, respectively.

Following resection, patients were longitudinally followed every 3 to 4 months for the first 2 years postoperative, every 6 months for years 2 to 5, and yearly for 5 to 10. Follow-up included a physical examination, cross sectional pulmonary imaging, and local imaging including an MRI of the surgical bed. All surviving patients had at least 2 years of clinical follow-up with a mean follow-up of 8 years (range 2 to 26).

### Statistical Analysis

Descriptive statistics were used to summarize the data. Continuous variables were reported as means ± standard deviations or medians with interquartile ranges, depending on distribution. Categorical variables were compared using Fisher’s exact tests. Local recurrence-free survival, metastasis-free survival, and disease-specific survival (DSS) were analyzed utilizing the Kaplan–Meier method with 95% confidence intervals. Cox proportional hazard analysis evaluated the risk for complications and recurrence. Statistical significance was set at a two-tailed *p* value of <0.05.

For the purposes of evaluating the performance of intraoperative frozen sections, the following definitions were used: “true positives” were considered to be patients who had R1 or R2 margins on both the frozen and permanent sections for the originally submitted specimen; “false positives” were considered to be patients who had R1 margins on the frozen section and R0 margins on the permanent pathology for the originally submitted specimen prior to re-excision; “true negatives” were considered to be patients who had R0 margins on both the frozen and permanent sections without subsequent local recurrence; “false negatives” were considered to be patients who had R0 margins on the frozen section and had R1 or R2 margins on the permanent section and/or developed local recurrence. Because of the way in which the frozen sections are cut, the permanent section rather than the frozen section represents the true final pathological margin. Sensitivity, specificity, positive predictive value, negative predictive value, and accuracy of the frozen section were calculated using the above. To additionally compare the intraoperative frozen section directly with the gold standard of the permanent pathology, accuracy was also separately re-calculated without consideration of local recurrence in the definitions of true and false negatives.

## 3. Results

### 3.1. Oncologic Outcomes

Over the course of the follow-up, 76 patients were alive and without signs of the disease, 27 patients died of causes other than myxofibrosarcoma, 8 patients died of disease, and 1 is currently alive with the disease. The 2-, 5-, and 10-year disease-specific survival rates were 93%, 93%, and 91%, respectively. There was no difference in the 10-year disease-specific survival (*p* = 0.461) between patients with a low grade (89%), intermediate grade (92%), and high-grade (90%) tumor. No analyzed factor was found to be associated with death due to the disease (Table 2).

Over the course of the study, disease recurrence occurred in 21 patients. Of these patients, twelve developed isolated metastatic disease, seven developed local recurrence, and two developed local recurrence and metastatic disease.

**Figure 1:**
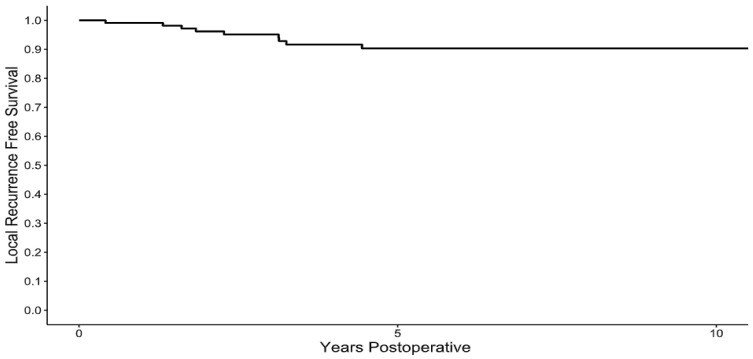
Local recurrence-free survival. Following resection of a superficial myxofibrosarcoma, the 1-, 2-, 5-, and 10-year local recurrence survival rates were 99%, 96%, 90%, and 90%.

Following resection, no local recurrences occurred after 5 years of clinical follow-up, with the 1-, 2-, 5-, and 10-year local recurrence-free survival rates of 99%, 96%, 90%, and 90%, (Figure 1), respectively. The median time to local recurrence was 26 months (ranging from 5 to 54). There was one case of a patient with an intermediate grade sarcoma which recurred as a high-grade sarcoma, otherwise the grade of the sarcoma did not change between the resected specimen and the local recurrence. In the review of risk factors for the development of local recurrence, patients who had a positive intraoperative frozen margin (HR 7.44, 95% CI [1.85–29.94], *p* = 0.004) and those with a final positive margin (HR 8.53, 95% CI [1.76–41.24], *p* = 0.007) were at risk for the development of local recurrence (Table 3). All local recurrences occurred in patients with high- or intermediate-grade sarcomas. There was no difference (*p* = 0.654) in the 5-year local recurrence-free survival rates between patients with a low- (100%), intermediate- (90%) or high-grade (88%) sarcoma. Local recurrence was not associated with death due to the disease (HR 9.36, 95% CI 0.53–163.49, *p* = 0.125). With all-cause death as a competing risk, the 5-year cumulative incidence of local recurrence was 8% (95% CI 4–16%). To manage the local recurrences, repeat excision was performed in eight patients, and one patient with widespread metastases was observed.

**Figure 2:**
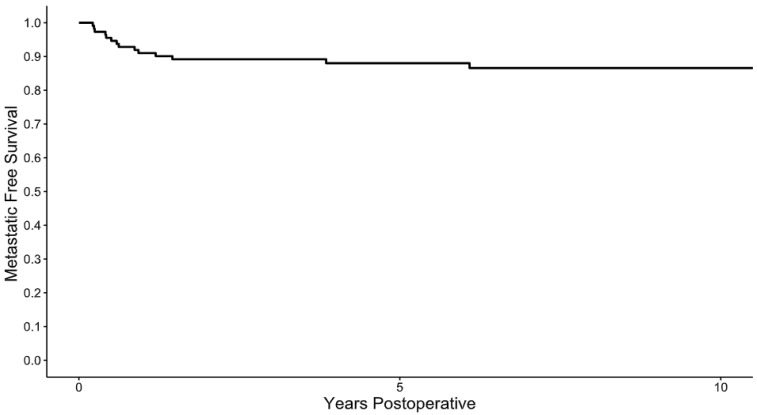
Metastatic free survival. Following resection of a superficial myxofibrosarcoma, the 2-, 5-, and 10-year metastatic free survival rates were as 89%, 88%, and 86%.

Following resection, the 2-, 5-, and 10-year metastasis-free survival rates were 89%, 88%, and 86% (Figure 2), respectively. Factors associated with metastatic disease included initial margins that were positive on frozen section analysis (HR 5.14, 95% CI 1.61–16.42, *p* = 0.005) and patients with a tumor > 5 cm (HR 3.22, 95% CI 1.01–10.29, *p* = 0.047). Of the 14 patients who developed metastatic disease, 9 developed metastases involving regional lymph nodes, of which 5 had isolated nodal disease. These five patients underwent resection of their nodal disease. In the patients with isolated nodal disease, one died of other causes at 33 months, one died of disease progression at 69 months, and the other three patients were alive at 24 months, 63 months, 20 years post-metastasectomy.

### 3.2. Surgical Outcomes

Intraoperative frozen section margins were negative in 103 (92%) patients. In these 103 patients, 1 patient had a reported “negative margin” but a positive margin on the permanent section. Of the nine patients whose frozen section analysis reported a microscopically positive margin, seven underwent immediate re-excision to a microscopically negative margin on frozen section analysis. In the additional two cases, one patient received postoperative radiotherapy, and one had close clinical observation due to the inability to remove additional tissue. Both patients developed a local recurrence.

Overall, there was 1 false positive, 7 false negatives, 9 true positives, and 96 true negatives. This included one false positive in a patient with a history of a previous inadvertent excision and radiotherapy where a scar was misidentified as “atypical cells concerning for tumor”; false negatives included one patient with a reported negative on the frozen section and positive on the final pathology, and six patients with a negative frozen margin and negative final margin on the permanent section with a subsequent local recurrence; true positives included six patients who underwent an immediate re-excision to negative margin with no local recurrence with positive margin on the frozen and final margin analysis, and two patients with a positive frozen section and positive final margin which could not be excised to a negative and developed a local recurrence; true negatives included ninety-six patients with negative frozen and final margins, and no local recurrence. The sensitivity for frozen section was 56.25% (95% CI 29.88–80.25%), the specificity was 98.97% (95% CI 94.39–99.97%), the positive predictive value was 90% (95% CI 57.98–98.51%), the negative predictive value was 93.20% (95% CI 88.72–95.98%), and the accuracy was 92.92% (95% CI 86.53–96.89%). However, when comparing frozen pathology directly to permanent pathology without considering subsequent local recurrence, the accuracy of frozen section analysis was 98.23% (95% CI 93.75–99.78%).

Definitive wound closure at the time of resection included pedicled or rotational flap coverage (*n* = 53, 47%), primary closure (*n* = 27, 24%), split thickness skin grafting (*n* = 20, 18%), and free flap (*n* = 12, 11%). Six patients had vacuum dressing placed to prepare the wound bed for skin grafting. All these patients had a negative margin on frozen section analysis, and the staged reconstruction was to optimize the skin grafting rather than having to wait for the final margin assessment.

### 3.3. Complications

Postoperative complications occurred in 34 patients (30%), most commonly a wound complication (*n* = 22, 20%) and infection (*n* = 15, 13%). Complications resulted in a total of 26 additional surgical procedures in 18 (16%) total patients. Seven of these patients had more than one additional surgical procedure. There were no repeat surgical procedures after 5 years postoperative. The 1-, 2-, and 5-year reoperation free survival rates were 84%, 84%, and 83%. With death as a competing risk, the cumulative incidence of reoperation at 5 years was 16%. The most common indication for a repeat surgical procedure was secondary to a wound complication (*n* = 25, 12%). One patient who initially underwent a skin graft following brachytherapy needed an additional free flap to salvage the wound. There were no cases of amputation.

## 4. Discussion

Myxofibrosarcoma is a highly infiltrative soft-tissue sarcoma, typically growing diffusely along vascular and fascial planes, making it difficult to achieve a negative margin. The results of the current study highlight the ability to perform a single-stage resection and reconstruction at a sarcoma center with a multidisciplinary team. This approach led to a low rate of local recurrence, negating the need for staged surgical resections. This treatment algorithm potentially saves patients additional unnecessary anesthetics, lowering overall cost and minimizing delays in the patient’s recovery.

Due to the infiltrative nature of myxofibrosarcoma, microscopically positive margins can occur in up to 25% of cases [15,16]. As a result, some institutions have employed wound VAC temporization to delay definitive closure until final margin status is confirmed [8,9,10,11]. The largest retrospective study, to date, examined 53 patients with superficial myxofibrosarcomas, finding a reduction in LR among the VAC group. However, the statistically significant importance of this is questionable since the authors only reported a one-tailed *p* value, and the follow-up time was shorter for the VAC group when compared to the single-stage group. This is important because the mean time to local recurrence was similar to the reported follow-up time for the VAC group [8]. These patients also underwent, on average, at least one additional surgical and anesthetic procedure (100% reoperation rate) which was potentially unnecessary, exposing patients to increased surgical and medical risks and causing increased financial costs to the health care system compared to an immediate wound closure [8]. Due to this, without a true potential benefit in the reduction in local recurrence based on the current published data, it is difficult to recommend this as a “paradigm” shift in the treatment of patients with a myxofibrosarcoma as the authors have recommended.

Intraoperative frozen section analysis, with the availability of sarcoma pathologists to review the specimen, is essential for our management of these patients [14,17,18]. At our institution, frozen section analysis has demonstrated a historical accuracy of 97.8%, with a clinically significant error rate of 0.1% [14]. Depending on how “false negatives” are defined, the accuracy of intraoperative frozen section analysis for myxofibrosarcoma is similar to our institutional historical accuracy for all tumors. This method allows for reliable margin assessment at the time of surgery; however, it is not available at all institutions. The way our margins are assessed are also different than how other centers routinely perform frozen margin assessments. Other centers have relied on “scouting” peripheral margins which has shown limited utility and is associated with increased cost [19]. At our center, all margins are assessed on the resected specimen. The specimen is inked and reviewed by the orthopedic oncologist and pathologist to determine where margins should be taken. This includes the closest margins and wider peripheral margins based on the preoperative MRI. Although newer methods to assess margins intraoperatively are being developed, this technology is still under investigation [20]. Until progress is made in the use of other modes of intraoperative margin assessment, the results of the current study highlight the importance of intraoperative frozen margin analysis in patients with infiltrative soft-tissue sarcomas. Similar results have been demonstrated in breast cancer patients treated at our institution, where intraoperative frozen section analysis resulted in improved oncologic outcome and fewer second surgeries [21].

A negative surgical margin is the main oncologic factor controlled by the surgeon and is paramount to local control. Obtaining a negative margin can be difficult in patients with myxofibrosarcoma as these tumors always have microscopic infiltration along fascial margins. Infiltration on preoperative MRI, the “tail sign”, has been associated with an elevated risk of local recurrence when not accounted for with the surgical resection [4,22,23,24]. Radiotherapy can assist in margin control in these patients as the fascial tails are included when planning radiotherapy. At our institution the use of radiotherapy in these patients is individualized. In cases where a local recurrence would not compromise limb-salvage, and there is the ability to obtain a wide margin with surgical resection alone, we have avoided preoperative radiotherapy for patients with small (<5 cm), superficial, low-grade myxofibrosarcomas.

Our selection bias to perform a resection alone in this low-risk group likely accounts for the fact that the use of radiotherapy was not associated with a reduction in local recurrence. Other centers have relied on postoperative radiotherapy, however the rate of local recurrence with postoperative radiotherapy can be high [2,25]. At our center we have utilized preoperative radiotherapy as this is an important adjuvant to improve local control [17,25,26]. In the current study, even with preoperative radiotherapy and a negative margin, the risk of local recurrence was elevated in patients where the initial frozen margin and final permanent margin were positive, which is similar to previous studies highlighting the importance of an initial negative margin resection in patients with myxofibrosarcoma [25]. The local biological aggressiveness of myxofibrosarcoma is highlighted by having the highest rate of local recurrence compared to other types of sarcomas, even with radiotherapy [5]. In the current series, this difficulty in achieving a negative frozen section margin translated into a higher risk of metastatic disease, again highlighting the possibility for a biologically aggressive tumor.

This study has limitations inherent to its retrospective design, which limits the data we were able to collect, and the analysis performed. As data was collected from existing records, we were limited by the completeness and accuracy of the documentation, which may introduce information bias. This study only looks at superficial myxofibrosarcoma and as such may not translate to other soft-tissue sarcoma histologies or deep myxofibrosarcoma. Additionally, the records were authored by multiple providers over an extended period, introducing variability in documentation standards, clinical practice, and diagnostic criteria. Likewise, multiple surgeons performed surgical procedures; however, the goals of the surgical resection were similar in all cases. This heterogeneity may contribute to information bias and limit the consistency of data extraction.

## 5. Conclusions

Overall, our institution does not employ wound vacuum-assisted closure (VAC) temporization for myxofibrosarcoma. Instead, management is directed by a multidisciplinary team of sarcoma and reconstructive specialists implementing a preoperative protocol aimed at optimizing both oncologic and reconstructive outcomes, which yields excellent outcomes and may be considered a preferred approach in experienced centers. Through this coordinated approach, definitive reconstruction occurs at the time of resection without compromising surgical margins or delaying wound closure. This process has demonstrated effective local control and acceptable rates of postoperative complications and unplanned reoperations, further supporting the efficacy of this approach.

## Figures and Tables

**Table 1:** Patients undergoing wide local excision of superficial myxofibrosarcoma.

Demographic	Patients (Total %)
Males	62 (55%)
Females	50 (45%)
Mean Patient Age	70 ± 14 Years
Previous Inadvertent Excision	70 (63%)
**Tumor Location**	
Lower Extremity	63 (57%)
Upper Extremity	41 (36%)
Trunk	8 (7%)
**Wound Closure Technique**	
Primary	27 (24%)
Split Thickness Skin Graft	20 (18%)
Pedicled/Rotational Flap	53 (47%)
Free Flap	12 (11%)

**Table 2:** Factors associated with death due to the disease.

Analyzed Factor	Hazard Ratio (95% CI)	*p* Value
Male	0.26 (0.05–1.33)	0.108
Previous Inadvertent Excision	1.64 (0.32–8.16)	0.545
Chemotherapy	1.77 (0.21–14.39)	0.593
Age > 70 Years	3.27 (0.65–16.37)	0.147
Tumors > 5 cm	3.99 (0.82–19.92)	0.091
Development of Local Recurrence	9.36 (0.53–163.49)	0.125

**Table 3:** Factors associated with tumor recurrence.

Analyzed Factor	Local Recurrence Hazard Ratio (95% CI)	*p* Value	Metastatic Disease Hazard Ratio (95% CI)	*p* Value
Male Sex	0.90 (0.24–3.36)	0.877	0.58 (0.20–1.68)	0.320
Previous Inadvertent Excision	1.94 (0.40–9.37)	0.401	1.44 (0.45–4.61)	0.533
Radiotherapy	0.94 (0.19–4.54)	0.942	1.72 (0.38–7.70)	0.475
Chemotherapy	3.84 (0.79–18.52)	0.09	0.93 (0.12–7.12)	0.945
Age > 70 years	2.32 (0.58–9.31)	0.23	2.67 (0.83–8.56)	0.096
Initial Positive Frozen Margin	7.44 (1.85–29.94)	0.004	5.14 (1.61–16.42)	0.005
Final Margin Positive	13.39 (2.74–65.38)	0.001	2.52 (0.33–19.33)	0.371
Tumor > 5 cm	1.69 (0.45–6.32)	0.43	3.22 (1.01–10.29)	0.047

## Data Availability

Data will not be shared.
